# Exogenous Melatonin Counteracts NaCl-Induced Damage by Regulating the Antioxidant System, Proline and Carbohydrates Metabolism in Tomato Seedlings

**DOI:** 10.3390/ijms20020353

**Published:** 2019-01-16

**Authors:** Manzer H. Siddiqui, Saud Alamri, Mutahhar Y. Al-Khaishany, M. Nasir Khan, Abdullah Al-Amri, Hayssam M. Ali, Ibrahim A. Alaraidh, Abdulaziz A. Alsahli

**Affiliations:** 1 Department of Botany and Microbiology, College of Science, King Saud University, Riyadh 2455, Saudi Arabia; saualamri@ksu.edu.sa (S.A.); muthr20@yahoo.com (M.Y.A.-K.); 438106173@student.ksu.edu.sa (A.A.-A.); hayhassan@ksu.edu.sa (H.M.A.); ialaraidh@ksu.edu.sa (I.A.A.); aalshenaifi@ksu.edu.sa (A.A.A.); 2 Department of Biology, Faculty of Science, University of Tabuk, Tabuk 71491, Saudi Arabia; nasirmn4@gmail.com

**Keywords:** SOD-CAT pathway, ASC-GSH pathway, melatonin, Δ^1^-pyrroline-5-carboxylate synthetase, *Solanum lycopersicum*, antioxidant system, proline, carbohydrate

## Abstract

Melatonin, a natural agent, has multiple functions in animals as well as in plants. However, its possible roles in plants under abiotic stress are not clear. Nowadays, soil salinity is a major threat to global agriculture because a high soil salt content causes multiple stresses (hyperosmotic, ionic, and oxidative). Therefore, the aim of the present study was to explore: (1) the involvement of melatonin in biosynthesis of photosynthetic pigments and in regulation of photosynthetic enzymes, such as carbonic anhydrase (CA) and ribulose-1,5-bisphosphate carboxylase/oxygenase (Rubisco); (2) the role of melatonin in osmoregulation by proline and carbohydrate metabolism; and (3) the function of melatonin in the antioxidant defense system under salinity. Outcomes of the study reveal that under non-saline conditions, application of melatonin (20 and 50 µM) improved plant growth, viz. shoot length, root length, shoot fresh weight (FW), root FW, shoot dry weight (DW), root DW and leaf area and physio-biochemical parameters [chlorophyll (Chl) *a* and *b*, proline (Pro) and total soluble carbohydrates (TSC) content, and increased the activity of CA and Rubisco]. However, tomato seedlings treated with NaCl exhibited enhanced Chl degradation, electrolyte leakage (EL), malondialdehyde (MDA) and reactive oxygen species (ROS; superoxide and hydrogen peroxide). ROS were detected in leaf and root. Interestingly, application of melatonin improved plant growth and reduced EL, MDA and ROS levels through upregulation of photosynthesis enzymes (CA, Rubisco), antioxidant enzymes (superoxide dismutase, catalase, glutathione reductase and ascorbate reductase) and levels of non-enzymatic antioxidants [ascorbate (ASC) and reduced glutathione (GSH)], as well as by affecting the ASC—GSH cycle. Additionally, exogenous melatonin also improved osmoregulation by increasing the content of TSC, Pro and Δ^1^-pyrroline-5-carboxylate synthetase activity. These results suggest that melatonin has beneficial effects on tomato seedlings growth under both stress and non-stress conditions. Melatonin’s role in tolerance to salt stress may be associated with the regulation of enzymes involved in photosynthesis, the antioxidant system, metabolism of proline and carbohydrate, and the ASC—GSH cycle. Also, melatonin could be responsible for maintaining the high ratios of GSH/GSSG and ASC/DHA.

## 1. Introduction

Large areas of the world, particularly arid and semi-arid regions, are severely affected by soil salinity. Elevation in heat stress increases the levels of NaCl in the soil which disturbs the water use efficiency (due to hyperosmotic stress) of plants. Prolonged exposure of plants to NaCl induces dual osmotic and ionic stress which trigger overproduction of reactive oxygen species (ROS) that cause extensive cellular damage, and dysfunction of physiological and molecular mechanisms of plants [1,2,3,4]. The continuous accumulation of salt in soil has been shown to be destructive to world agriculture by severely reducing crop production. Due to salinity, crop performance and production are well below their genetic capacity [5,6]. The intensity of salinity effects varies from plants to plants and species to species, and also depends on the concentration of NaCl in the soil, sensitivity of crops and genetic makeup of crops for salt tolerance. Thus, it is necessary to explore innovative techniques to improve crop performance by transforming morphology, and physiological and biochemical mechanisms. 

Melatonin (M; *N*-acetyl-5-methoxytryptamine), a low-molecular-weight organic compound, acts as a pleiotropic signaling molecule, and plays vital roles in both animal and plants by regulating many physiological processes [7,8,9]. In animals, it was identified as a hormone, which regulates multiple biological processes, such as antioxidant activity, immunological enhancement, circadian rhythms, sexual behavior, sleep physiology, seasonal reproductive physiology and temperature homeostasis [10,11,12,13,14]. However, in plants, it was first reported in 1995 [15,16]. Thereafter, additional studies confirmed that M was present in the organs of several plant species, such as seeds, roots, leaves, stems, and fruits [9,17]. M has been established as a novel class of metabolic regulator in the biological kingdom [18]. It plays essential roles during seed germination, growth and development, root growth, photosynthesis, leaf senescence, flowering and in the regulation of fruit ripening and production of fruits quality [7,17,19,20,21,22,23]. Also, M behaves as a hormone as well as an antioxidant molecule and plays an important function in tolerance of plants to abiotic stresses [24,25,26,27]. The modulation of endogenous M levels in plant tissue has been shown to induce root development, mitosis, and mitotic spindle formation [18]. In many studies, it has been reported that exogenous M promotes seed germination and seedlings growth, and also induces the regulation of growth-related genes expression involved in cell wall growth and expansion [28,29]. M triggers the photosynthetic activity by up-regulating the expression of genes (*PsaA*, *PsaF*, *PsaG*, *PsaH*, *PsaK*, and *PsaO* in photosystem I, and *PsbE*, *PsbO*, *PsbP*, *PsbQ*, *PsbY*, *PsbZ*, and *Psb28* in photosystem II) [30]. Additionally, M stimulates the sucrose biosynthesis pathway by regulating the sucrose-related enzymes and genes [31]. It maintains the photosynthetic process by inhibiting degradation of chlorophyll and proteins, and regulating metabolism of sugar and nitrogen [20,22]. M interacts with calcium and ROS signaling networks, as well as with auxin signaling, and is also involved in other metabolic pathways [20]. 

Earlier M has been established to be a beneficial molecule that regulates the many biological processes in animals. Many studies have shown that M acts as an antioxidant and plays an important role in protecting plants against different environmental stresses, including stress caused by heat [26], salt [32,33,34], heavy metals [35], UV-B radiation [36] and drought [37]. One of the important roles of M is to detoxify the ROS by generating free radicals scavenging-cascade and activating antioxidant enzymes [19,26,38,39,40]. M may be involved in the regulation of most stress signaling transduction pathways in receptor-dependent or independent manners and in the expression of genes involved in plant tolerance to abiotic stresses [24,26]. Also, a comprehensive study on *Arabidopsis* at the genetic level in the response of exogenous application of M confirmed that M alters a large number of genes related to stress tolerance and upregulates transcript levels for many stress receptors and the most genes in the salicylic acid, jasmonic acid, abscisic acid, and ethylene pathways [41,42]. These results clearly indicate the critical roles of M in the tolerance of plants to different biotic and abiotic stresses [41]. Although the function of M in the protection of plants against different environmental stresses by improving defense system has been extensively studied [26,32,33,34,35,36,37], the detail mode of action of M against salt stress by improving enzymes activity involved in the photosynthesis process in plants is still fragmentary.

Tomato (*Solanum lycopersicum* L.) is a well-known vegetable crop. Worldwide, tomato is the second most consumed vegetable (following potato consumption). Tomatoes and tomato products are good sources of nutrients and antioxidant properties that help in reducing the risks of cardiovascular disorder and cancer. The tolerance to salinity differs greatly across the various species in the plant kingdom. Like other crops, salt stress significantly produces an inhibitory effect on every aspect of physiology and biochemistry of tomato plant [43,44]. Therefore, it is necessary to understand the physiological and biochemical mechanisms of tomato plants to improve tolerance to salt stress through the exploitation of different cultural practices. To establish the role of M in the tolerance of tomato plants to salinity, the present study was performed to examine: (1) the involvement of M in biosynthesis of photosynthetic pigments and in regulation of photosynthetic enzymes, such as carbonic anhydrase (CA) and ribulose-1,5-bisphosphate carboxylase/oxygenase (Rubisco) (2) the involvement of M in osmoregulation by proline and carbohydrate metabolism, and (3) the function of M in antioxidant system under salinity.

## 2. Results

### 2.1. Effect of Melatonin on Growth Attributes of Tomato Seedlings under NaCl Stress

**Figure 1:**
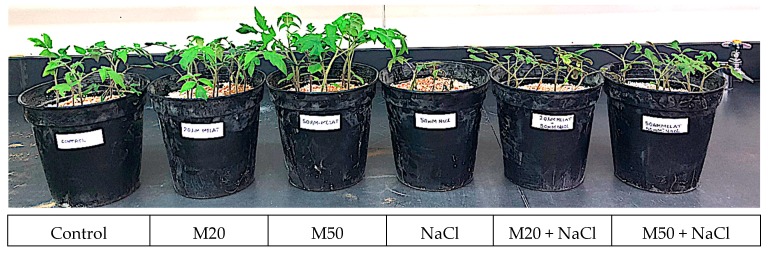
Growth performance of tomato seedlings under melatonin and salinity conditions.

To understand the involvement of M in growth under salt stress, we measured shoot length (SL), root length (RL), shoot fresh weight (FW), root FW, shoot dry weight (DW), root DW, and area per leaf (LA) of tomato seedlings under salinity (Table 1 and Table 2, and Figure 1). Tomato seedlings that received 50 µM M (M50) showed marked differences in morphology as compared to the seedlings treated with 20 µM M (M20) and also control plants (Table 1 and Table 2). Under salinity, seedlings received M (M20 and M50) showed better morphology than NaCl-treated plants (Figure 1). Also, this could be seen with the recorded results for growth characteristics (SL, RL, shoot FW, root FW, shoot DW, root DW, and LA) of tomato seedlings. Under non-stress conditions, application of 20 µM and 50 µM of M (M20 and M50) improved SL by 21.36% and 39.22%, RL by 32.75% and 83.11%, shoot FW by 27.54% and 41.30%, root FW by 111.48% and 155.74%, shoot DW by 16.90% and 29.14%, root DW 101.10% and 124.44%, and LA by 10.13% and 36.06%, respectively, as compared to control plants. The results presented in Table 1 and Table 2 show that salinity adversely affected SL, RL, shoot FW, root FW, shoot DW, root DW, and LA of tomato plants. However, application of M at the rate of 20 and 50 µM (M20 and M50) enhanced all these parameters under NaCl stress (Table 1 and Table 2). It was observed that 50 µM of M (M50) was more effective than 20 µM (M20) in protecting plants from salinity, as reflected by higher values of all the studied growth parameters under salt stress conditions (Table 1 and Table 2). Under salt stress, application of 20 and 50 µM of M (M20 and M50) improved SL by 23.85 and 37.93%, RL by 26.24 and 48.19%, shoot FW by 52.11 and 88.73%, root FW by 92.86 and 121.42%, shoot DW by 50.01 and 73.55%, root DW by 52.76 and 100.16%, and LA by 87.96 and 115.64%, respectively as compared with the plants treated with NaCl only (Table 1 and Table 2).

### 2.2. Effect of Melatonin on Chl Content, and Activities of CA and Rubisco under NaCl Stress

**Figure 2:**
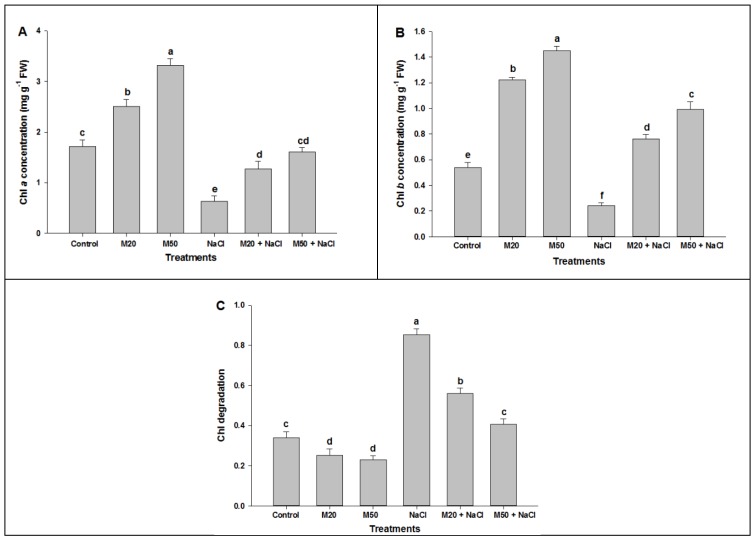
Effect of melatonin on (**A**) Chl *a*, (**B**) Chl *b* and (**C**) Chl degradation in leaves of tomato seedlings under salinity. Data represent mean of 4 replicates with bars indicating SE. The bars labelled with different letters are significantly different at *p* < 0.05%. [DDW (control), 20 µM melatonin (M20), 50 µM melatonin (M50), 100 mM NaCl (NaCl)].

To explore the role of M in the biosynthesis of photosynthesis pigments and the activity of enzymes involved in photosynthesis under stress and non-stress conditions, we estimated the concentration of chlorophyll (Chl) *a* and *b*, and activity of CA and Rubisco (Figure 2). Biosynthesis of Chls is an important marker in determining the salt tolerance capacity of plants. The perusal of the data exhibits that salinity caused a substantial reduction in the levels of Chl *a* and Chl *b*. NaCl treated plants exhibited the lowest values for all studied parameters as compared with M and control (Figure 2A,B). In contrast, the highest levels of Chl *a* and Chl *b* were observed in non-stressed plants supplied with 50 µM of M (M50). Similar results were also observed in salt-suffered tomato seedlings which showed a significant improvement in Chls content when treated with M. It was observed that under salinity, 50 µM of M (M50 + NaCl) proved better than 20 µM of M (M20 + NaCl) in improving the concentration of Chl *a* and Chl *b* (Figure 2A,B). Figure 2C shows that seedlings treated with NaCl exhibited degradation in Chl content. However, this degradation was reduced when the seedlings were treated with M (M20 and M50).

**Figure 3:**
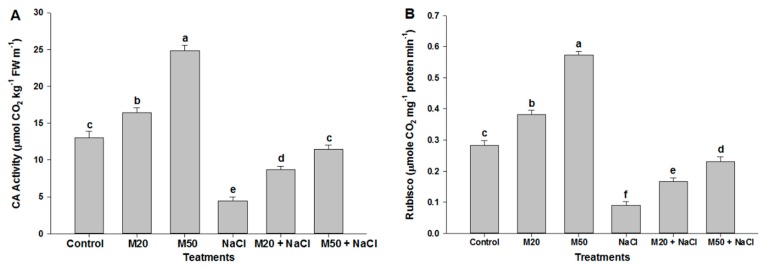
Effect of melatonin on (**A**) carbonic anhydrase (CA) and (**B**) ribulose-1,5-bisphosphate carboxylase/oxygenase (Rubisco) activity in leaves of tomato seedlings under salinity. Data represent mean of 4 replicates with bars indicating SE. The bars labelled with different letters are significantly different at *p* < 0.05%. [DDW (control), 20 µM melatonin (20M), 50 µM melatonin (50M), 100 mM NaCl (NaCl)].

Two important enzymes involved in photosynthesis i.e., carbonic anhydrase (CA) and ribulose-1,5-bisphosphate carboxylase/oxygenase (Rubisco) increased with increasing concentrations of M. Seedlings that received 50 µM of M (M50) exhibited maximum activity of these enzymes as compared to 20 µM of M (M20) under non-stress conditions (Figure 3A,B). However, plants under salinity exhibited less activity of CA and Rubisco enzymes than the control plants. Application of M at the rate of 20 µM (M20) and 50 µM (M50) enhanced the activity of these enzymes under salt stress conditions (Figure 3A,B). The results show that a higher concentration of M (M50) proved to be more effective than its lower concentration (M20) in relieving the inhibitory effect of salt stress (Figure 3A,B).

### 2.3. Effect of M on Electrolyte Leakage (EL) and Malondialdehyde (MDA) Concentration under NaCl Stress

**Figure 4:**
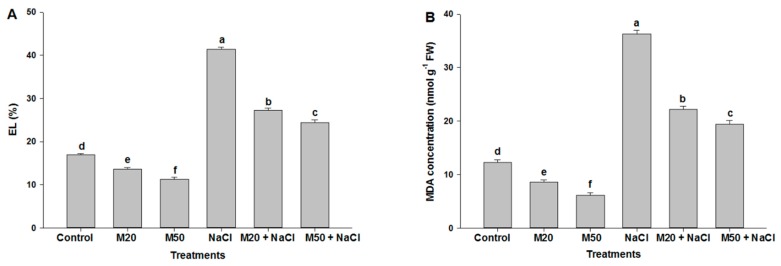
Effect of melatonin on (**A**) electrolyte leakage (EL), and (**B**) malondialdehyde (MDA) content in leaves of tomato seedlings under salinity. Data represent mean of 4 replicates with bars indicating SE. The bars labelled with different letters are significantly different at *p* < 0.05%. [DDW (control), 20 µM melatonin (20M), 50 µM melatonin (50M), 100 mM NaCl (NaCl)].

The effects of salinity on membrane permeability and peroxidation of membrane lipids were assessed by measuring EL (%) and MDA concentration, respectively. Exposure of plants to 100 mM NaCl caused more than a two-fold increase in EL and MDA content over the control plants. However, the smallest values were recorded in non-stressed plants treated with 50 µM of M (M50). A significant reduction in EL and MDA concentration was also noticed in salt-suffered plants treated with 20 µM M (M20) or 50 µM M (M50) as compared with the plants treated with NaCl only (Figure 4A,B). Furthermore, 50 µM of M (M50) was found to be more effective than 20 µM of M (M20) in suppressing salt stress, as shown by a greater reduction in EL and MDA levels by 50 µM of M (M50) (Figure 4A,B).

### 2.4. Effect of Melatonin on Hydrogen Peroxide (H_2_O_2_) and Superoxide (O_2_^•−^) Concentration under NaCl Stress

**Figure 5:**
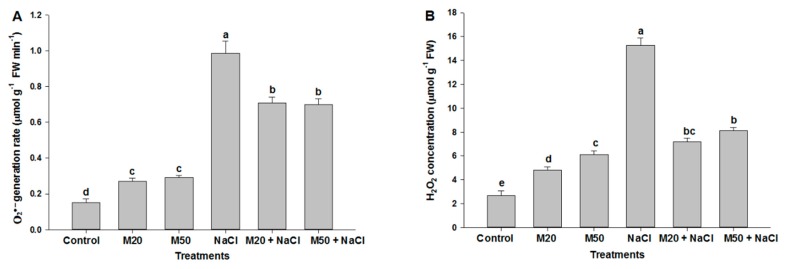
Effect of melatonin on (**A**) O_2_^•−^ content, and (**B**) H_2_O_2_ content in leaves of tomato seedlings under salinity. Data represent mean of 4 replicates with bars indicating SE. The bars labelled with different letters are significantly different at *p* < 0.05%. [DDW (control), 20 µM melatonin (20M), 50 µM melatonin (50M), 100 mM NaCl (NaCl)].

**Figure 6:**
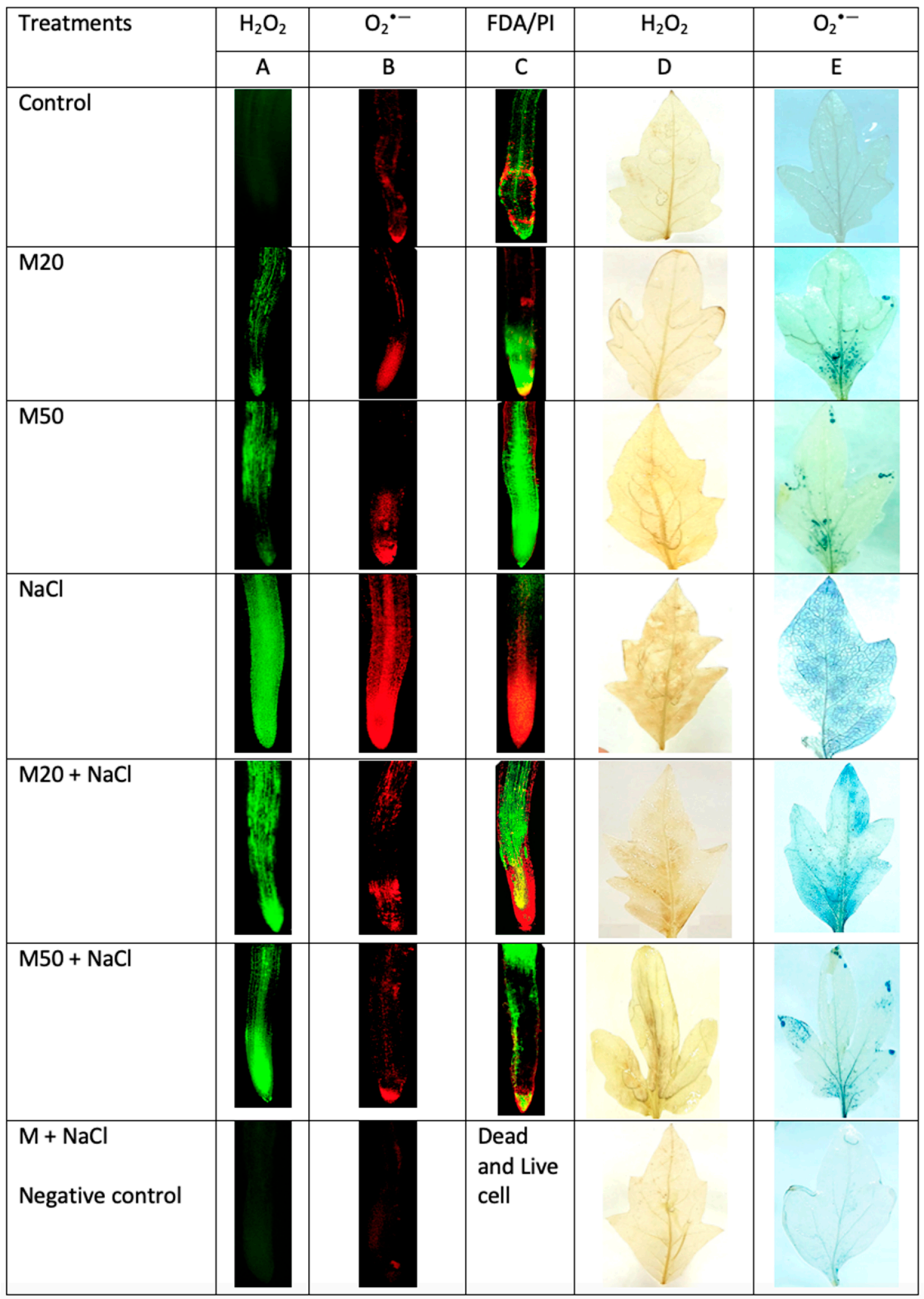
Under fluorescence microscope imaging of ROS, (**A**) H_2_O_2_ production (H_2_O_2_-dependent DCF-DA fluorescence) in root, (**B**) O_2_^•−^ production (O_2_^•−^ dependent DHE fluorescence) in root, (**C**) Overlay projection image of root stained with FDA (green: viable cells) and PI (red: non-viable cells), (**D**) H_2_O_2_ production in leaf using DAB and (**E**) O_2_^•−^ formation in leaf using NBT staining under NaCl and melatonin application. As negative controls, roots and leaves of NaCl and melatonin exposed plants were preincubated with ascorbic acid (1 mM ASC), a H_2_O_2_ scavenger and tetramethyl piperidinooxy (1 mM TMP), an O_2_^•—^ scavenger. [DDW (control), 20 µM melatonin (20M), 50 µM melatonin (50M), 100 mM NaCl (NaCl)].

To investigate the role of M in inhibiting the over-production of ROS under salt stress, we determined the formation of H_2_O_2_ and O_2_^•−^ in root and leaf of tomato seedlings (Figure 5 and Figure 6). The results given in Figure 5A,B reveal that salinity induced oxidative stress occurred through generating ROS (H_2_O_2_ and O_2_^•−^) in tomato plants. The recorded concentration of H_2_O_2_ and O_2_^•−^ under NaCl stress was the highest among all the treatments. However, salt-stressed plants treated with M showed a substantial reduction in the concentration of studied ROS (Figure 5A,B). Furthermore, as shown in Figure 5A,B, both levels of M i.e., 20 µM (M20) and 50 µM (M50) showed statistically similar results under salinity. To evaluate the visual effect of M and NaCl on the production of ROS in root and leaf of tomato seedlings, in situ formation of H_2_O_2_ and O_2_^•−^ was visualized in roots using a DCF-DA and DHE fluorescence probes, respectively. The effect of M on the production of H_2_O_2_ and O_2_^•−^ in leaves under NaCl stress was also assessed using DAB and NBT staining, respectively. After exposure of tomato seedlings to NaCl, a sharp increase in green and red fluorescence signal was detected in the roots compared with the control, and the plants treated with M (M20 and M50) (Figure 6A,B). Similarly, leaves of NaCl treated seedlings showed a sharp brown and blue color as compared to controls, and seedlings treated with both the levels of M under stress and non-stress conditions. In this experiment, a similar result was observed for dead and live cells using double propidium iodide (PI) and fluorescein diacetate (FDA) staining. The highest number of dead cells was visualized (red) in the roots of NaCl-stressed seedlings as compared to the control and M treated-seedlings (Figure 6C). At the same time, seedlings treated with M (20 and 50 µM of M) exhibited maximum live cells (green signal) as compared to NaCl stressed-seedings. 

### 2.5. Effect of Melatonin on Proline (Pro) Content, Δ^1^-Pyrroline-5-Carboxylate Synthetase (P5CS) Activity and Total Soluble Carbohydrates (TSC) Content under NaCl Stress

**Figure 7:**
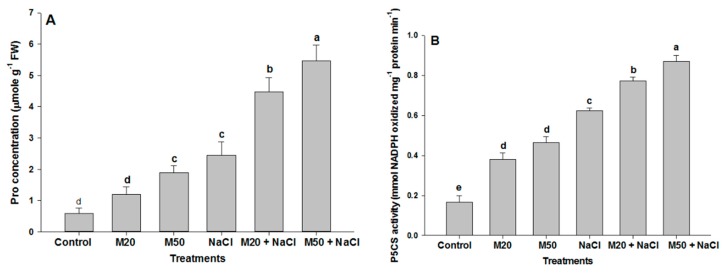

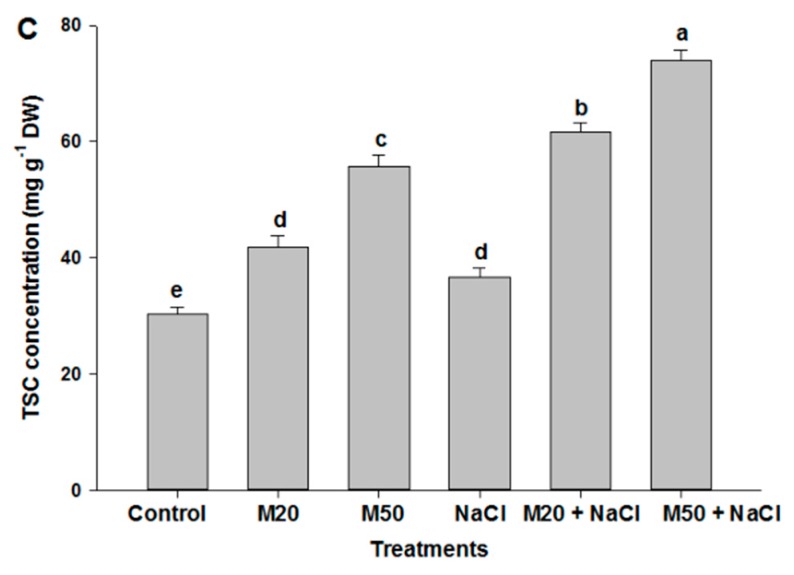
Effect of melatonin on (**A**) proline (Pro) content, (**B**) Pyrroline-5-carboxylate synthase (P5CS) activity and (**C**) total soluble carbohydrates (TSC) content in leaves of tomato seedlings under salinity. Data represent mean of 4 replicates with bars indicating SE. The bars labelled with different letters are significantly different at *p* < 0.05%. [DDW (control), 20 µM melatonin (20M), 50 µM melatonin (50M), 100 mM NaCl (NaCl)].

To further explore the mechanism of M involved in Pro biosynthesis and TSC accumulation, we analyzed changes in the activity of enzyme P5CS and TSC content in the leaves of tomato seedlings treated with M and NaCl. Exposure of tomato seedlings to 100 mM NaCl enhanced Pro content, P5CS activity and TSC content as compared with their respective controls (Figure 7A–C). In addition, a further increase in these parameters was recorded when salt-stressed seedlings were treated with 20 and 50 µM of M (M20 + 100 mM NaCl and M50 + 100 mM NaCl) as compared with salt stressed seedlings. Moreover, stressed as well as non-stressed seedlings treated with 50 µM M (M50 and M50 + 100 mM NaCl) showed the higher level of Pro content, P5CS activity and TSC content than the seedlings treated with 20 µM M (M20 and M20 + 100 mM NaCl) (Figure 7). 

### 2.6. Effect of Melatonin on Non-Enzymatic Antioxidants under NaCl Stress

**Figure 8:**
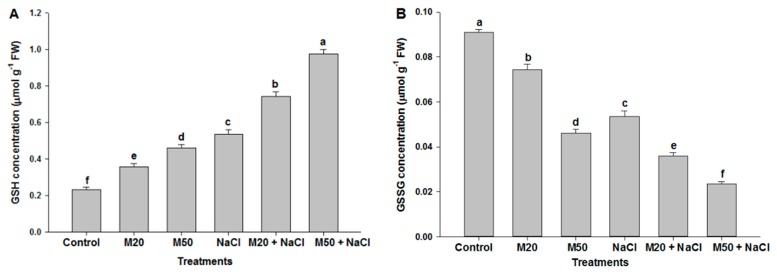

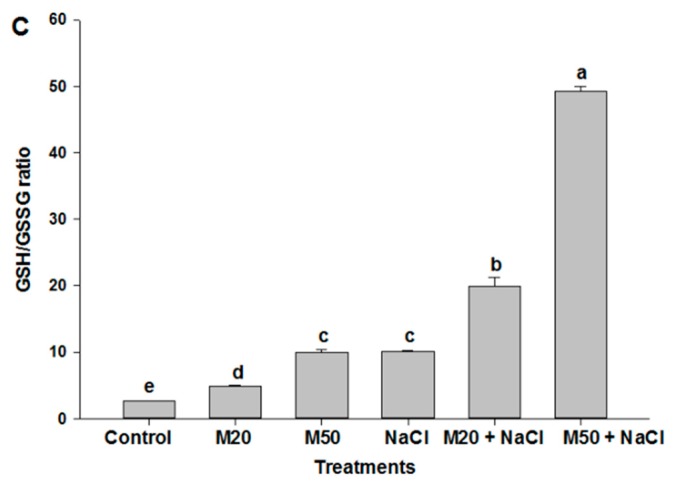
Effect of melatonin on the content of (**A**) reduced glutathione (GSH), (**B**) oxidized glutathione, and (**C**) ratio of GSH/GSSG in leaves of tomato seedlings under salinity. Data represent mean of 4 replicates with bars indicating SE. The bars labelled with different letters are significantly different at *p* < 0.05%. [DDW (control), 20 µM melatonin (20M), 50 µM melatonin (50M), 100 mM NaCl (NaCl)].

**Figure 9:**
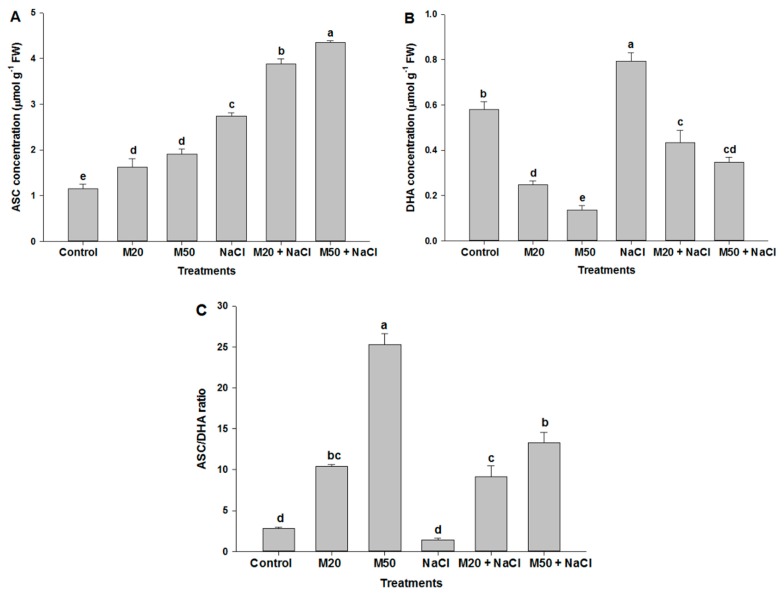
Effect of melatonin on the content of (**A**) ascorbate (ASC), (**B**) dehydroascorbate (DHA), and (**C**) ratio of ASC/DHA in leaves of tomato seedlings under salinity. Data represent mean of 4 replicates with bars indicating SE. The bars labelled with different letters are significantly different at *p* < 0.05%. [DDW (control), 20 µM melatonin (20M), 50 µM melatonin (50M), 100 mM NaCl (NaCl)].

To elucidate the potential role of M in tolerance of seedlings to salt stress, we determined non-enzymatic antioxidants accumulation. The results showed that salinity significantly enhanced the concentration of non-enzymatic antioxidant reduced glutathione (GSH), whereas a decrease in oxidized glutathione (GSSG) concentration was recorded under salt stress as compared with the control (Figure 8A,B). At the same time an increase in the GSH/GSSG ratio was noted in NaCl treated plants (Figure 8C). However, the application of both levels of M increased the concentration of GSH under stress as well as non-stress conditions; in contrast, the reverse was true for GSSG concentration. The effect of M on GSH and GSSG concentration was also reflected in the GSH/GSSG ratio, which was at the highest value with the application of 50 µM M (M50 + 100 mM NaCl) (Figure 8C). Effect of NaCl and M on non-enzymatic antioxidants was studied by estimating the concentration of ascorbate (ASC), dehydroascorbate (DHA) and the ASC/DHA ratio (Figure 9A–C). Plants exposed to NaCl showed an increase in the concentration of ASC and DHA compared with the control (Figure 9A,B). Application of both levels of M enhanced ASC content under stressed as well as non-stressed conditions. Salt-stressed plants treated with 50 µM M (M50 + 100 mM NaCl) exhibited higher level of ASC than the same stressed plants treated with 20 µM M (M20 + 100 mM NaCl) as compared with the plants treated with NaCl alone (Figure 9A). Regarding DHA content, both levels of M decreased DHA content in the salt-stressed plants; moreover, M at the rate of 50 µM caused more reduction in DHA concentration than 20 µM as compared with the salt-stressed plants (Figure 9B). It is evident from Figure 9C that salt stress caused the smallest reduction in the ASC/DHA ratio among all the treatments. However, plants treated with 50 µM M had the highest ratio of ASC/DHA under both stressed and non-stressed conditions (Figure 9C).

### 2.7. Effect of Melatonin on the Activities of Antioxidant Enzymes under NaCl Stress

**Figure 10:**
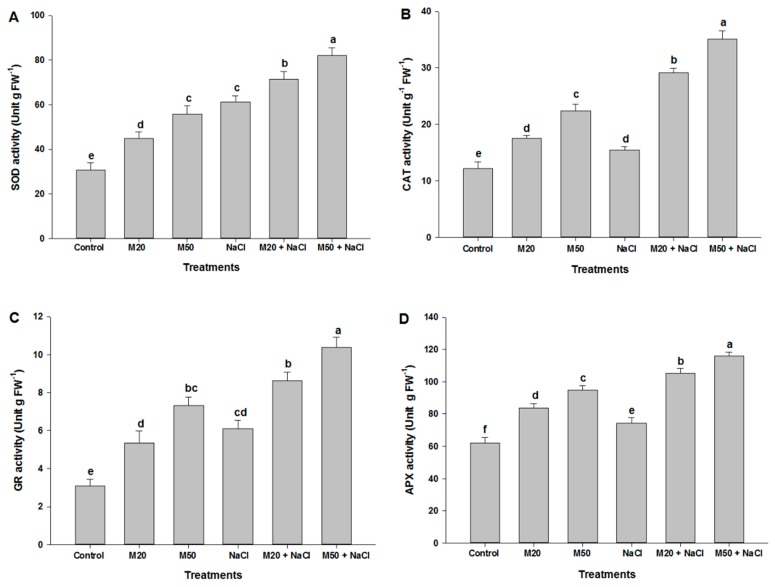
Effect of melatonin on the activities of (**A**) superoxide dismutase (SOD), (**B**) catalase (CAT), (**C**) glutathione reductase (GR) and (**D**) ascorbate peroxidase (APX) in leaves of tomato seedlings under salinity. Data represent mean of 4 replicates with bars indicating SE. The bars labelled with different letters are significantly different at *p* < 0.05%. [DDW (control), 20 µM melatonin (20M), 50 µM melatonin (50M), 100 mM NaCl (NaCl)].

To further assess the mechanism of M-induced tolerance to salinity, we examined changes in the regulation of antioxidant enzymes activities. From the results presented in Figure 10 it is apparent that NaCl-imposed plants exhibited increased activity of superoxide dismutase (SOD), catalase (CAT), glutathione reductase (GR) and ascorbate peroxidase (APX) as compared with their respective controls. Application of M to salt-stressed seedlings (M20 + 100 mM NaCl and M50 + 100 mM NaCl) further enhanced the activity of these enzymes compared with the salt-stressed seedlings not treated with M, although the treatment 50 µM M (M50) proved more effective in enhancing the activity of antioxidant enzymes in stressed as well as non-stressed plants. Stressed plants treated with 50 µM M (M50 + 100 mM NaCl) showed the highest activities of SOD, CAT, GR and APX compared to the remaining treatments (Figure 10A–D).

## 3. Discussion

### 3.1. Exogenous Melatonin Enhances Growth by Stimulating Photosynthetic Enzymes, and Proline and Carbohydrate Metabolism under Salinity and Non-Salinity Conditions

It is well known that the plant, a sessile organism, fights against different abiotic stresses through their developed defense mechanisms. Under environmental stress, M is formed endogenously in different parts of plants (root, leaf, fruit, and seed) and regulates several physiological and biochemical functions in plants. In this study, under stress and non-stress conditions, the beneficial and defensive roles of M were unveiled. The obtained results reveal that M had a significant response on the plant growth under non-salinity conditions, (Figure 1, and Table 1 and Table 2). Also, under salinity conditions, application of M significantly improved growth characteristics, such as SL, RL, shoot FW, root FW, shoot DW, root DW, and LA. In both conditions, the effect of M was found to be dose dependent; application of 50 µM M (M50) gave the highest values for all the above the mentioned growth parameters as compared to 20 µM M (M20). These results contradict the findings of Shi et al. [45] who reported that M had the significant effects on growth and physiological parameters of bermudagrass only under abiotic stress (salt, drought and freezing); its effects were non-significant under normal conditions. However, the results of the present study substantiate the findings of Murch and Saxena [46]; Hernandez-Ruiz et al. [47] and Wei et al. [30]. Under normal conditions, M promotes root growth and vegetative growth of plants [30,48]. An increase in SL and RL of tomato seedlings may be due to the role of M in physiological functions similar to indole-3-acetic acid (IAA), because M has the same precursor as IAA [48]. Additionally, M increases endogenous levels of free IAA in roots. Most of the studies reported that M mimics the role of IAA and is able to increase SL and RL, and induces root generation and also promotes the formation of new lateral and adventitious roots [13]. Under salinity, the protective role of M can also be explained on the basis of its ameliorating function in increasing growth characteristics by inducing the activity of antioxidant enzymes and the levels of non-enzymatic antioxidants (Figure 1, Figure 8, Figure 9 and Figure 10; Table 1 and Table 2). In addition to the beneficial role of M under non-stress condition, it was also be involved in the restoration of altered plant growth characteristics suppressed by NaCl stress (Figure 1). M-induced enhancement in SL and RL might have helped the seedlings to bear a greater number of well-oriented leaves for better harvesting of solar energy [49,50]. All these factors together instigated the plants for more carbon fixation that resulted in improved FW and DW of the treated plants (Table 1 and Table 2). However, additional studies are needed to unveil the physiological and molecular mechanisms through which M influences plant growth and development.

In plants, photosynthesis is one of the important key physiological processes which determine plant growth and development, and is responsible for dry matter production. Like other physiological, biochemical, and molecular processes, the process of photosynthesis is affected by different environmental stresses [51]. In this study, results show that exogenous application of M exhibited a beneficial effect on Chls content by lowering Chl degradation under non-stress condition (Figure 2A–C), it may be due to its role in the synthesis of porphyrins, glycine and succinyl-CoA by regulating d-aminolevulinate synthase activity [52]. However, NaCl stress reduced the photosynthetic pigments (Chl *a* and *b*) synthesis and increased Chl degradation (Figure 2A–C); possibly due to an accumulation of sodium ions that may cause changes in fine structure of chloroplast [53,54] and also alters instability of pigments protein complexes, resulting in Chl reduction and degradation (Figure 2A–C). Interestingly, from these results, it is quite clear that treatment M (M20 and M50) may have restored altered photosynthetic pigments synthesis induced by salinity (Figure 2A,B). Moreover, under salinity, M increases ferredoxin which regulates the synthesis of reduced GSH and shields Chl from degradation [30,55]. The results are consistent with a recent study on melon showing that exogenous M maintained Chl stability under cold stress [56]. 

Earlier studies reported that M played a beneficial role in the regulation of plant growth and development under salinity [32,57,58]. The present study also confirms the beneficial effect of M on plant growth of tomato seedlings under both salinity and non-salinity conditions (Figure 1, and Table 1 and Table 2), and role of M in the regulation of enzymes activity, such as CA and Rubisco. It is well documented that both CA and Rubisco are important enzymes that are involved in carbon fixation during photosynthesis. The enzyme CA, a zinc-metalloenzymes, is required for the reverse conversion of CO_2_ and HCO_3_^−^, and CO_2_ is fixed by the enzyme Rubisco. CA enzyme has many physiological roles, such as exchange of ions, maintenance of acid base balance, carboxylation/decarboxylation reactions and facilitation of CO_2_ diffusion across the chloroplast membranes [59,60]. Also, Rubisco is the key enzyme involved in the first step of carbon assimilation. In the present study, salinity suppressed the activity of CA and Rubisco in tomato seedlings that did not receive M (M20 and M50) (Figure 3A,B). This may be due to destabilization in the folding configuration of many native proteins, causing an inhibition of enzyme activity [61] and resulting in the inhibition of plant growth (Table 1 and Table 2). However, application of M (M20 and M50) significantly induced the activity of these enzymes (Figure 3A,B), which might be responsible for maintaining a constant supply of CO_2_ to the plants during photosynthesis by improving photosynthetic pigments (Chl *a* and *b*) (Figure 2A,B). Thus, we suggest that M was not only involved in plant growth; it also improves the tolerance of tomato seedlings to salt stress by activating photosynthetic enzymes. 

We know that during the normal cellular metabolism, ROS are generated by oxidative reaction process of mitochondrial respiration and photosynthesis process. This might be the reason for increased ROS in M-treated seedlings under normal conditions (Figure 5 and Figure 6). At low amounts, ROS show positive effects, and act as signaling molecules during repairing processes of cells [62]. On the other hand, onset of cellular oxidative damage is the hallmark of salt stress which is indicated by the lipid peroxidation, EL, and the content of H_2_O_2_ and O_2_^•−^ (Figure 4, Figure 5 and Figure 6). The levels of oxidative damage were measured by MDA, H_2_O_2_, O_2_^•−^ and EL in leaves and roots of tomato seedlings treated with 100 mM NaCl a (Figure 4, Figure 5 and Figure 6). These results reveal that salinity may have caused cell death by enhancing lipid peroxidation (Figure 6C). These results corroborate the findings of previous studies that high salinity causes ROS formation by NADPH oxidase accumulation [2,63]. However, M supplied seedlings exhibited reduced oxidative damage by inhibiting the overproduction of these ROS and MDA as well as EL, which were also observed in leaves and roots of seedlings via a microscope (Figure 6A–E). This may be due to the role of M in plants under abiotic stress, as it acts as an antioxidant and upregulates the expression of antioxidant coding genes/enzymes, thereby reduced formation of ROS [30]. M reacts with ROS, resulting in the formation of M-derivatives that makes M even more capable in the detoxification of ROS, even at low levels [64,65,66] because M can easily cross cellular boundaries and protect the biological system by regulating the metabolic flow of thiol-compounds, such as reduced GSH (Figure 8A) [67]. Furthermore, the obtained results of the present study show that the application of M improved contents of Pro and TSC, and the activity of antioxidant enzymes and non-antioxidant (Figure 7A,C, Figure 9A,B and Figure 10A–D); these factors together resulted in reduced oxidative damage by scavenging ROS. 

Under abiotic stress, plants accumulate organic solutes in the cytosol and organelles where they function as osmolytes and regulate physiological processes in plants [68,69]. Also, even Pro is present in small amount and plays multiple roles, such as stabilization of membrane and proteins, redox homeostasis and regulation of salt stress-responsive genes’ expression [70,71]. In the present study, seedlings fed with NaCl and M exhibited higher levels of Pro and TSC as compared to the control (Figure 7A,C). However, M supplied to stressed-seedlings further accelerated the synthesis of Pro and TSC. An increase in Pro and TSC content may be due to an increase in the activity of P5CS, and Rubisco and CA under both stress and non-stress conditions, respectively (Figure 3A,B and Figure 7B). An M-mediated increase in Pro and TSC may confer improved salt stress tolerance of tomato seedlings because Pro may reduce ROS and maintain GSH redox state (Figure 8), and TSC as major soluble constituents that provide energy and carbon skeletons that help cells to grow rapidly and synthesize required organic molecules [13,72,73,74]. Therefore, this work indicates that improved tolerance of tomato seedlings to salt stress may be associated with the accumulation of Pro and TCS induced by M application.

### 3.2. Exogenous Melatonin Regulates Antioxidant System under Salinity 

As is known from earlier studies that under different environmental stresses, plants efficiently counter oxidative stress and maintain redox homeostasis by a series of ROS-scavenging systems orchestrated by non-enzymatic and enzymatic antioxidant mechanisms [75,76]. During detoxification, the antioxidant enzyme SOD is used as the first line of defense, which dismutates O_2_^•−^ to O_2_ and H_2_O_2_, whereas CAT removes H_2_O_2_ by reducing H_2_O_2_ to H_2_O. APX is a vital constituent of the ASC-GSH cycle and catalyzes the conversion of H_2_O_2_ to H_2_O in cytosol and chloroplast where ASC acts as a reducing agent by providing electron. Also, GR is a crucial enzyme involved in Halliwell and Asada pathways (ASC-GSH). Meanwhile, the non-enzymatic antioxidants (GSH, ASC and DHA) play key roles in regulating the antioxidant enzymes activities, maintaining normal cellular functions, thereby increasing tolerance of plants to abiotic stress. In the present experiment, seedlings treated with NaCl had an increase in the content of GSH, ASC and DHA as compared to control (Figure 8 and Figure 9). The ratio of GSH/GSSG in the cell is one of the important determinants for oxidative stress when the seedlings were exposed to oxidative stress due to the formation of ROS (Figure 5 and Figure 6) by NaCl stress the accumulation of GSSG was increased, and GSH and the ratio of GSH/GSSG were decreased (Figure 8A–C). Interestingly, the application of M maintained the ratios of GSH/GSSG and ASC/DHA by increasing the content of ASC and GSH and by decreasing the content of DHA and GSSG in tomato seedlings. Besides the crucial role of M as a potent antioxidant, it also acts as a first line of defense against abiotic stress [25] and also maintains redox homeostasis by promoting high Pro and TSC accumulation (Figure 7A,C) [77]. M also plays a key role in the upregulation of antioxidant enzymes involved in ASC-GSH pathway, which might have enabled tomato seedlings to cope with oxidative stress and prevented cell damage or death (Figure 6C) caused by overproduction of ROS due to salinity. Studies of bermudagrass grown under cold stress [26], *Malus hupehensis* under salinity [32], tomato under salt stress [58], found that exogenous application of M induced the ASC-GSH cycle by activating enzymes and non-antioxidant enzymes which were responsible for tolerance of pants to environmental stress. Our studies indicate that reduced cellular damage and cell death (Figure 6C) by exogenous supply of M was closely linked to improved ROS detoxification by the involvement of SOD, CAT, GR, APX and ASC-GSH pathway. However, in this experiment, a decrease in DHA content under salinity was recorded which might have been due to its use in the synthesis of the ASC with the help of GSH and thiol as an electron donor [78]. M induced tolerance of tomato seedlings may be associated with the favorable ratio of reduced and oxidized forms of redox compounds (GSH/GSSG and ASC/DHA). 

## 4. Materials and Methods 

### 4.1. Plant Materials and Culture Conditions

To achieve the objectives, the experiment was conducted in a growth chamber, King Saud University, Riyadh, Kingdom of Saudi Arabia, where the light intensity (250 μmol of photons m^−2^ s^−1^), photoperiod (16/8-h light/dark), temperature (25 ± 3 °C) and relative humidity (65%–75%) were maintained. Tomato (*S. lycopersicum* L. cv. Five Star) seeds were germinated in plastic pots (12 cm diameter) filled with a mixture of acid-washed-sand and peat (1:1) after seeds sterilization in a solution (49% sterile DDW), 50% ethanol and 1% sodium hypochlorite). At the first true stage, the equal size seedlings of tomato were transferred to the pots filled with a mixture of acid-washed-sand and peat (1:1). Pots were organized in a simple randomized design with four replicates per treatment. After establishment of 4 seedlings in each pot, the following applications of M and NaCl were applied to each experimental pot: (i) 0 µM M (M) + 0 mM NaCl (control), (ii) 20 µM M + 0 mM NaCl, (iii) 50 µM M + 0 mM NaCl, (iv) 0 µM M + 100 mM NaCl, (v) 20 µM M + 100 mM NaCl and (vi) 50 µM M + 100 mM NaCl. The above treatments were supplied to the plants with half-strength Hoagland’s nutrient solution for every two days. To avoid osmotic shock, NaCl treatment was applied gradually until the desired concentration was achieved. The seedlings were exposed to the above treatments for 25 days. The included concentration of M and NaCl in the present experiment was based on the earlier studies of Hasan et al. [79], and Siddiqui et al. [2], respectively.

### 4.2. Morphological Characteristics of Tomato Seedlings Determination of Tomato Seedlings

After 25 days of sowing, the seedlings were collected for morphological, physiological and biochemical analysis. All treated-seedlings were separated into roots, stems and leaves for measuring SL, RL, shoot FW, root FW, shoot DW, root DW and LA. For DW and after that, samples were kept in an oven at 70 °C for 48 h for dry weight. Leaf Area Meter (LI-3050A, LI-COR Inc., Lincoln, NE, USA) was used to measure the area of three leaves (upper, middle, and lower) of each seedling of tomato.

### 4.3. Physiological and Biochemical Characteristics Analysis of Tomato Seedlings

#### 4.3.1. Photosynthetic Pigments 

Fresh leaf samples were collected, and extraction was done using DMSO for determining of chlorophyll (Chl) *a* and *b* [80]. The content of Chls in the extract was read using a UV–vis spectrophotometer (SPEKOL 1500; Analytik Jena AG, Jena, Germany).

Leaf extract was used to measure the Chl degradation using spectrophotometrically and expressed as the ratio of absorbance (435/415) [81].

#### 4.3.2. Photosynthetic Enzymes 

Fresh leaf samples from each treatment were used to determine the activity of carbonic anhydrase (CA) and Rubisco was assayed according to the methods of Dwivedi and Randhawa [82], and Usuda [83], respectively. The activity of CA was expressed as μMol (CO_2_) kg^−1^ (FW) s^−1^. The activity of Rubisco was determined by estimating the oxidation of NADH at 340 nm, and expressed in μmol CO_2_ fixed min^−1^ mg^−1^ protein. The quantification of protein was determined [84].

#### 4.3.3. Cell Membrane Stability, Lipid Peroxidation, and ROS Determination and Detection 

To estimate the cell membrane stability, EL in fully expanded leaves of seedlings was measured according to the procedure explained by Lutts, et al. [85]. To determine the level of lipid peroxidation, the final product i.e., malondialdehyde (MDA) content was estimated according to the method of Heath and Packer [86]. In leaf samples, an extraction was made using a solution containing 10% trichloroacetic acid (TCA) and 0.65% 2-thiobarbituric acid. The content of MDA in leaf sample was presented as nmol g^−1^, FW.

Endogenous H_2_O_2_ was extracted from leaves using a solution of 5% TCA following the modified method of Velikova, et al. [87]. Superoxide was estimated in leaf following the modified method of Elstner and Heupel [88].

Detection of H_2_O_2_ and O_2_^•−^ in root was done using fluorescence probes 2′,7′-dichlorofluorescein diacetate (DCF-DA) and dihydroethidium (DHE) respectively. In root, H_2_O_2_ and O_2_^•−^ were visualized according to the method described by Rodriguez-Serrano, et al. [89]. The signals of DCF-DA and DHE were captured using a fluorescence microscope (Eclipse Ni-U, Nikon, Tokyo, Japan) at the excitation and emission wavelengths of (480 and 530 nm) and (490 and 520 nm), respectively. 

In leaf, H_2_O_2_ and O_2_^•−^ were detected following the methods described by Wang, et al. [90] and Mostofa and Fujita [91], respectively. Blue insoluble formazan for O_2_^•−^ and a deep brown polymerization product for H_2_O_2_ were detected, and images were taken using a camera.

Under salinity, the viability of root cells was monitored using the methods described by Fan et al. [92] and Jones, et al. [93]. For dual staining of root cells, a solution of FDA and PI was prepared by mixing FDA (2 µL from the stock solution (1g FDA/mL) with PI (10 µL from the stock solution (2 µg PI/mL) and final volume of 1 mL was made up with DDW. The tips of each root were incubated in a double staining solution for 10 min at room temperature in darkness. The images were taken using a fluorescence microscope at excitation and emission wavelengths of 488 nm and 630 nm, respectively. 

#### 4.3.4. Determination of Pro Content and Its Metabolizing Enzyme P5CS and Total Soluble Carbohydrates 

Estimation of proline (Pro) in seedlings of tomato was conducted according to the ninhydrin method described by Bates et al. [94]. In order to determine the activity of P5CS involved in proline metabolism, the extraction was performed according to the method of Sumithra et al. [95] and determined according to the method of Charest and Phan [96]. The activity of P5CS was measured by monitoring the rate of consumption of NADPH by the decrease in absorbance at 340 nm.

Seedlings were collected and used for the determination of total soluble carbohydrates (TSC) by taking the absorbance at 490 nm, using glucose as a standard. TSC was expressed as mg g^−1^ dry weight (DW) [97].

#### 4.3.5. Determination of Antioxidants and Antioxidant Enzymes Assay

Prior to performing the enzymes assay, seedling samples of each treatment were collected and homogenized and suspended in an ice-chilled extraction buffer [50 mM Tris−HCl, pH 7.8, 1 mM EDTA, 1 mM MgCl_2_ and 1.5% (*w*/*w*) polyvinylpyrrolidone]. To prepare the crude extract for the determination of ascorbate peroxidase (APX), 2 mM of ascorbate (ASC) was added. The homogenates were centrifuged at 13,000 rpm for 20 min at 4 °C and the supernatant was collected and used for the assay of enzymes activities, such as SOD, CAT, GR and APX. The supernatant was also used to measure the activity of SOD, CAT, GR and APX according to methods described by Beyer and Fridovich [98], Aebi [99], Foyer and Halliwell [100] and Nakano and Asada [101] respectively.

For estimation of ASC, DHA, GSH and GSSG, homogenization of leaf tissue (0.25 g) was performed in a mixture (2% (*m*/*v*) metaphosphoric acid, 2 mM EDTA) and thereafter centrifuged at 13, 000 rpm for 15 min at 4 °C. The supernatant was stored for the determination of these non-enzymatic antioxidants. A method described by Takahama and Oniki [102] followed to determine the content of ASC/DHA in tomato seedlings with some modification as explained by Turcsányi et al. [103]. After neutralization of the supernatant (50 µL) with 100 mM K-phosphate buffer (pH 6.1), initial absorbance for ASC content was assessed spectrophotometrically at 265, followed by a second reading taken by 1 unit of ascorbate oxidase. The content of DHA content was measured by taking initial absorbance (265 nm) as for ASC in another 50 μl supernatant. Thereafter, absorbance was remeasured following the addition of 2 mM DL-dithiothreitol. 

The estimation of GSH and GSSG content was carried out according to methods of Yu et al. [104] with modifications as described by Paradiso et al. [105]. For estimation of glutathione pool was performed using 0.3 mL of 0.5 M phosphate buffer (pH 7.5). After neutralization, the reaction was initiated by adding 3 unit of GR to a solution (0.2 mM NADPH, 100 mM phosphate buffer (pH 7.5), 5 mM EDTA, and 0.6 mM 5,5′dithiobis (2-nitrobenzoic acid) (DTNB). The reaction was evaluated by the rate of absorption changes at 412 nm of 2-nitro-5-thiobenzoic acid that was generated from the reduction of DTNB. GSSG was determined by adding 2-vinylpyridine (20 μL) to eliminate GSH, whereas of H_2_O_2_ (20 μL) was added for GSH assay. The concentration of GSH and GSSG was calculated from standard curves, in which GSH and GSSG were plotted against the rate of absorbance change at 412 nm. 

### 4.4. Statistical Analysis

The obtained data were subjected to analysis of variance (ANOVA) and expressed as the mean ± standard error of four independent replicates. Differences between treatment means were compared statistically by a Duncan’s multiple-range test at the level of *p* < 0.05 using SPSS Ver. 17 statistical software (SPSS Inc., Chicago, IL, USA).

## 5. Conclusions

**Figure 11:**
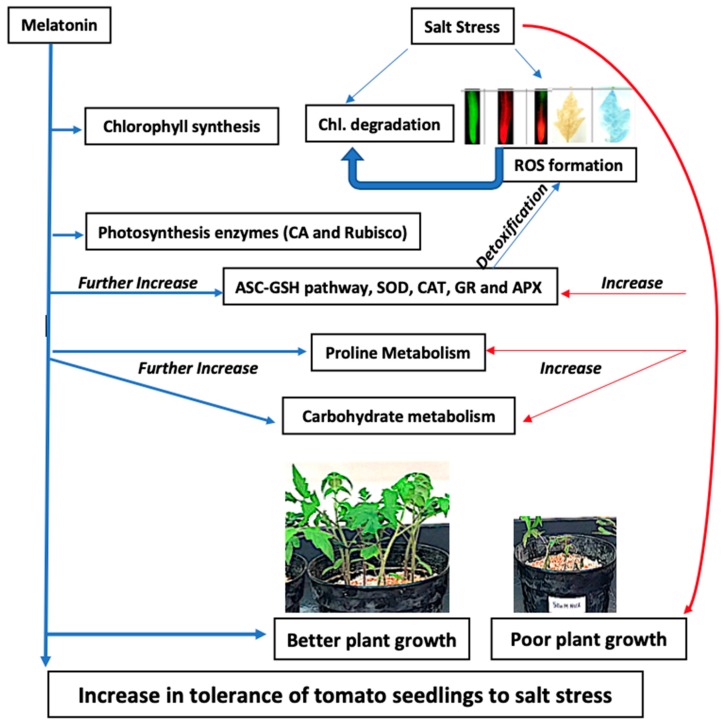
Summary of melatonin-induced tolerance against salinity by regulating the antioxidant system, proline and carbohydrate metabolism and photosynthetic pigments synthesis in plants. Ch-chlorophyll, ROS-reactive oxygen species, CA-carbonic anhydrase, ASC-ascorbate, GSH-reduced glutathione, SOD-superoxide dismutase, CAT-catalase, GR-glutathione reductase, APX-ascorbate peroxidase.

In conclusion, exogenous application of M significantly not only increased plant growth (SL, RL, shoot FW, root FW, shoot DW, root DW, and LA) by increasing physiological and biochemical parameters of tomato seedlings (Chl *a* and *b*, CA, Rubisco, Pro, P5CS and TSC) under normal condition but also conferred an improved tolerance of tomato seedlings to NaCl stress. NaCl supplied to tomato seedlings severely affected all of these parameters. As depicted in Figure 11, the application of M prevented a decrease in growth parameters under salinity by decreasing oxidative damage (reduced levels of H_2_O_2_ and O_2_^•−^ in leaf and root) and by an upregulation of antioxidant enzymes (SOD, CAT, GR and APX) and the ASC-GSH pathway which detoxified the levels of ROS (H_2_O_2_ and O_2_^•−^) in leaf and root. Subsequently, Chl degradation and cellular damage due to salinity were inhibited with the application of M as manifested by the increased Chl *a* and *b* and decreased MDA content as compared to NaCl-fed plants. This study also reveals that exogenous M may be responsible for improved resistance against salinity by activating the ROS scavenging system (enzymatic and non-enzymatic antioxidants), maintaining favorable ratios of ASC/DHA and GSH/GSSG, and also regulating proline and carbohydrate metabolism in tomato seedlings. This experiment, therefore, provides evidence that M acts as a plant growth regulator, and works as a potent elicitor against salt stress condition. 

## Figures and Tables

**Table 1:** Effect of melatonin on shoot length (SL), root length (RL), shoot fresh weight (shoot FW) and root fresh weight (root FW) of tomato seedlings (25 days old) under salinity (100 mM NaCl).

Treatments	Parameters			
	SL (cm)	RL (cm)	Shoot FW (g)	Root FW (g)
Control	20.32 ± 1.19 ^c^	6.87 ± 0.34 ^c^	1.38 ± 0.093 ^b^	0.061 ± 0.006 ^d^
M20	24.66 ± 0.90 ^b^	9.12 ± 0.17 ^b^	1.76 ± 0.093 ^a^	0.129 ± 0.003 ^b^
M50	28.29 ± 0.70 ^a^	12.58 ± 0.28 ^a^	1.95 ± 0.078 ^a^	0.156 ± 0.005 ^a^
NaCl	13.21 ± 0.59 ^e^	4.42 ± 0.14 ^e^	0.71 ± 0.067 ^d^	0.042 ± 0.006 ^e^
M20 + NaCl	16.36 ± 0.55 ^d^	5.58 ± 0.28 ^d^	1.08 ± 0.038 ^c^	0.081 ± 0.005 ^c^
M50 + NaCl	18.22 ± 0.64 ^cd^	6.55 ± 0.11 ^c^	1.34 ± 0.091 ^b^	0.093 ± 0.004 ^c^

Data followed by the same letters within a column are not significantly different at *p* < 0.05%. Average of four determinations is presented with ± SE.

**Table 2:** Effect of melatonin on shoot dry weight (shoot DW), root dry weight (root DW) and leaf area (LA) of tomato seedlings (25 days old) under salinity (100 mM NaCl).

Treatments	Parameters		
	Shoot DW (mg)	Root DW (mg)	LA (cm^2^)
Control	92.67 ± 2.33 ^c^	12.03 ± 0.58 ^c^	33.08 ± 0.53 ^c^
M20	108.33 ± 2.60 ^b^	24.19 ± 0.61 ^b^	36.43 ± 0.91 ^b^
M50	119.67 ± 2.02 ^a^	27.00 ± 0.58 ^a^	45.01 ± 0.58 ^a^
NaCl	45.33 ± 3.18 ^f^	6.33 ± 0.67 ^e^	10.55 ± 0.63 ^f^
M20 + NaCl	68.00 ± 1.73 ^e^	9.67 ± 0.67 ^d^	19.83 ± 0.77 ^e^
M50 + NaCl	78.67 ± 1.85 ^d^	12.67 ± 0.67 ^c^	22.75 ± 1.25 ^d^

Data followed by the same letters within a column are not significantly different at *p* < 0.05%. Average of four determinations is presented with ± SE. [double-distilled water (DDW) (control), 20 µM melatonin (M20), 50 µM melatonin (M50), 100 mM NaCl (NaCl)].

## Abbreviations

CA	Carbonic anhydrase
Rubisco	ribulose-1,5-bisphosphate carboxylase/oxygenase
Pro	Proline
TSC	Total soluble carbohydrates
MDA	Malondialdehyde
Chl	Chlorophyll
EL	Electrolyte leakage
GSH	Reduced glutathione
GSSG	Oxidized
CAT	Catalase
SOD	Superoxide dismutase
APX	Ascorbate peroxidase
ASC	Ascorbate
DHA	Dehydroascorbate reductase
